# The host response to influenza infections in human lung and macrophages cell lines

**DOI:** 10.1186/s12863-025-01341-2

**Published:** 2025-07-08

**Authors:** Carmen Aguilar, Ruth Lydia Olga Lambertz, Mark Heise, Ana Eulalio, Klaus Schughart

**Affiliations:** 1 Institute of Molecular Infection Biology (IMIB), University of Würzburg, Würzburg, Germany; 2 CJD Braunschweig, Braunschweig, Germany; 3 Department of Genetics, University of North Carolina at Chapel Hill, Chapel Hill, NC USA; 4 Department of Microbiology and Immunology, University of North Carolina at Chapel Hill, Chapel Hill, NC USA; 5 Center for Neuroscience and Cell Biology (CNC-UC), Centre for Innovative Biomedicine and Biotechnology (CIBB), University of Coimbra, Coimbra, Portugal; 6 Centre for Bacterial Resistance Biology (CBRB), Department of Life Sciences, Imperial College London, London, UK; 7 Institute of Virology Münster, University of Münster, Münster, Germany; 8 Department of Microbiology, Immunology and Biochemistry, University of Tennessee Health Science Center, Memphis, TN USA

**Keywords:** Influenza, Human, Lung cells, Macrophage, Transcriptome

## Abstract

**Objective:**

The innate immune response of an infected host is an essential defense mechanism to fight influenza virus infections in the respiratory tract. This response is essential to limit virus replication and spread. However, an exacerbated response may cause severe immune-pathologies. Therefore, it is very important to better understand innate immune responses at the level of its molecular networks in the context of viral infections.

**Data:**

We infected human lung adenocarcinoma (A549) and human monocytic (THP-1) cells with H3N2 influenza virus A virus and performed transcriptome analysis using next generation RNA sequencing at various times post infection. We report raw sequence data and normalized log_2_ transformed gene expression values. This data will allow researchers in the field to identify differentially expressed genes and pathways between the two cell types and over times post infection. Furthermore, our data enables comparisons to molecular studies performed in humans and animal models in the context of respiratory viral infections.

## Objective

Influenza virus infections represent a severe health problem with high morbidity and mortality worldwide [1]. During the early phase of an infection, the host activates a large number of inflammatory and antiviral responses, the so called innate immunity response [2, 3]. This defense system aims to restrict viral replication and to limit virus spreading in the respiratory tract [4, 5]. However, if the response is too strong or not well controlled, it may also result in severe immuno-pathologies [6–11]. It is therefore important to understand the biology and molecular interaction networks of the host repose during this early phase of infection. However, in human patients, it is only possible to analyze peripheral blood, or in some cases, broncho-alveolar lavages [12–28]. Therefore, studies at the level of infected lung epithelial cells and innate immune cells are essential to understand the molecular basis of the innate defense mechanisms. Here, we describe the host response in human cell lines infected with influenza A virus (IAV). We studied A549 cells, a human lung carcinoma cell, and THP-1 cells, a human monocyte cell line differentiated to macrophage-like cells, at early times after infection with H3N2 influenza A virus and performed detailed transcriptome studies during the early phase of infection.

## Data description

### Cells and virus

Human lung adenocarcinoma cells [29] and human monocytic cells [30] were infected with influenza virus A/Panama/2007/99 (H3N2) [31]. Virus was plaque purified on Madin-Darby canine kidney II (MDCK) cells (ATCC), then propagated in the chorio-allantoic cavity of 10-day-old specific pathogen free embryonated chicken eggs (Charles River Laboratories, Germany) for 48 h at 37 °C, aliquoted and stored at −80 °C, as described previously [32]. The titer of the stock viruses was determined by f.f.u. assay [33]. Viral RNA was extracted using the QiAmp Viral RNA Extraction Kit (Qiagen) and sequenced on Illumina MiSeq using MiSeq Reagent Kit v2 (500 cycles, paired end run). In contrast to the original published sequence [34], after plaque purification we observed three amino acid mutations: PB2: I411V; NP: L456V and M: N183S. We provide the fasta sequence for the virus genome (see Table 1 [35]). A549 and THP-1 cells were cultured separately in RPMI-1640 medium supplemented with 10% FBS. For in vitro infections, A549 cells (5.5 × 10⁴ cells/well) and THP-1 cells (1.8 × 10⁵ cells/well) were seeded in 24-well plates and incubated for 48 h to allow A549 cells to reach 90% confluence and for THP-1 cells to differentiate into macrophage-like cells following treatment with 50 ng/mL phorbol 12-myristate 13-acetate (PMA; P8139, Sigma-Aldrich).

### Infection

Cells were then infected with influenza A/Panama/2007/99 virus at a multiplicity of infection of 1 for A549 cells and 0.1 for THP-1 cells. Infection was carried out for 1 h at 37 °C. Briefly, cells were washed with PBS 1× Mg²⁺/Ca²⁺, containing 0.1% bovine serum albumin (BSA), followed by incubation with the virus in the same PBS buffer for 1 h at 37 °C. After incubation, cells were washed twice with the same buffer, and the infection medium was replaced with RPMI-1640 containing 0.1% BSA and freshly added 1.5 µg/mL TPCK-trypsin (4370285, Sigma-Aldrich). Infected cells were maintained at 37 °C until collection. Cells were harvested at 4, 8, and 24 h post-infection (p.i.) for A549 cells and at 4, 8, 12, and 24 p.i. for THP-1 cells. As controls, cells were treated in the same way as for infections, except that PBS was used without containing virus.

### RNA isolation and sequencing

Total RNA, including the small RNA fraction, was extracted using the miRNeasy Micro Kit (Qiagen, 217084) according to the manufacturer’s instructions. RNA integrity was assessed using the RNA Nano 6000 Assay Kit (Agilent Technologies, 5067 − 1511) on a Bioanalyzer 2100 system (Agilent Technologies), and RNA concentration was determined using a NanoDrop spectrophotometer (Thermo Scientific). One µg of RNA was used for strand-specific cDNA library construction using a NEB directional RNA lib prep kit (cat# E7420L, NEB, MA, USA) according to manufacturer’s protocol. Briefly, mRNA was enriched using oligo(dT) beads followed by two rounds of purification, and fragmented randomly by adding fragmentation buffer. The first strand cDNA was synthesized using random hexamer primers, after which a custom second-strand synthesis buffer (Illumina), dUTPs (incorporating dUTP in place of dTTP to create blunt-ended cDNA), RNase H and DNA polymerase I were added to generate the second-strand (ds cDNA). After a series of terminal repair, poly-adenylation, and sequencing adaptor ligation, the double-stranded cDNA library was completed following size selection and PCR enrichment. The resulting 250–350 bp insert libraries were quantified using a Qubit 2.0 fluorometer (Thermo Fisher Scientific, Waltham, MA, USA) and quantitative PCR. Size distribution was analyzed using an Agilent 2100 Bioanalyzer (Agilent Technologies, Santa Clara, CA, USA). Qualified libraries were sequenced on an Illumina HiSeq 4000 Platform (Illumina, San Diego, CA, USA) using a paired-end 150 run (2 × 150 bases). 40 M raw reads were generated from each library. Infections and subsequent processing of RNA and sequencing was done in the same batch for both cell lines, which allows direct comparison of responses in A549 and THP-1 cells.

### Bioinformatics

Reads from RNAseq were quality checked with FastQC (version 0.11.9 [36]), then trimmed using Trimgalore (version 0.6.7 [37]), with default settings. Trimmed reads were mapped to the human genome (ENSMBL hg38 release 111), normalized and log_2_-transfromed using STAR (version 2.5.2b [38]), with default settings. Mapped reads were counted at the gene level using RsubRead (version 1.32.4 [39]). Virus sequences were identified by mapping to the virus genome using the same protocol as above.

### Data

We confirmed the high quality and read depth of our data. On average, 46,010,393 reads were generated per sample, and 43,377,730 reads were mapped to the human genome (release 111) and 42,887,855 reads were assigned to annotated genes. Data are provided as raw sequence data (Table 1 [40]), and as normalized log_2_-transformed relative expression values. For the normalized expression matrix, deposited at GEO, raw reads were mapped to an earlier human genome version (release 91) and separately to the virus genome. Raw counts were then combined, normalized and log transformed (Table 1 [41]). In addition, we mapped the raw counts to a newer version of the human genome (release 111), but not to the virus genome and then normalized it (Table 1 [42]). Also, we provide a fasta sequence file for the virus that was use for the infections (Table 1 [35]). In addition, we provide a quality report (Table 1 [43]), and a description of samples with specific treatments and collection times (Table 1 [44]). To demonstrate the high quality and potential use of our data set, we performed identification of differentially expressed genes. Using DESeq2 (version 2.7.9a [45]), with thresholds log_2_ = 1 for log-fold change and adjusted *p*-value of > 0.05, we identified many DEGs for various contrasts [46].

Here, we report on the transcriptome responses in human lung cells and human macrophages after infection with H3N2 influenza A virus. The data allows identification of differentially expressed genes and pathways for both cell types at different times p.i. Furthermore, it will permit comparisons of the innate immune response in these two human cell lines widely used to study influenza infection, representative of lung (A549 cells) and professional phagocytic (THP-1) cells. To our knowledge, this is one of the most comprehensive analysis which has been performed for the innate immune responses in these two cell types. The results of our study will allow other researchers in the field to relate responses in these human lung and monocyte cells with responses in the blood of human patients and to studies that were performed in more complex systems, like organoids or lung slices. Furthermore, our data enables comparisons to the many molecular studies that have been performed in animal models, like mouse, ferrets, hamster or others.

**Table 1:** Overview of data files/data sets

Label	Name of data file/data set	File types (file extension)	DOI or accession number & reference
*Data file 1*	*Raw sequence files*	*fastq*	*Gene Expression Omnibus* https://identifiers.org/ncbi/insdc.sra:SRP311617 [40]
*Data file 2*	*Normalized expression values after mapping to human genome hg38 (ENSMBL hg38 release 91)*,* performed at the time of data submission to GEO*	*Text (txt)*	*Gene Expression Omnibus* https://identifiers.org/geo:GSE169309 [41]
*Data set 3*	*ST1_QC_030325.xlsx* *Quality control of raw reads before trimming*,* after trimming*,* uniquely mapped reads and percentage of mapped reads after mapping to human genome ENSMBL hg38 release 111*	*Excel*	*Figshare* 10.6084/m9.figshare.28524317.v1 [43]
*Data set 4*	*ST2_sample_characteristics_030325.xlsx* *Description of sample characteristics*	*Excel*	*Figshare* 10.6084/m9.figshare.28524356 [44]
*Data file 5*	*ST3_norm_A549_THP1_120225.txt* *Normalized expression values after mapping to human genome ENSMBL hg38 release 111*	*Text (txt)*	*Figshare* 10.6084/m9.figshare.28562609 [42]
*Data file 6*	*H3N2_pan99* *Sequence of A/Panama/2007/99 (H3N2) virus used for infections*	*fasta*	*Genbank* https://identifiers.org/ncbi/insdc:PV643427.1 [35]
*Data file 7*	*ST4 Number of differentially expressed genes for various contrasts*	*MS Word (docx)*	*Figshare* 10.6084/m9.figshare.29198843 [46]

### Limitations

Our study has some limitations. The experiments were performed in established cell lines derived from human tumors and may thus have acquired characteristics that do not fully represent the responses in normal lung tissues or monocytes/macrophages. The release of infectious particles was not monitored in this study. A correlation with the phase of replication, increasing or at peak production was thus not possible. 

## Data Availability

Raw transcriptome data and processed data, after mapping to human genome hg38 (ENSMBL hg38 release 91) and H3N2 virus are available at the public Gene Expression Omnibus [47] database (GEO-ID: GSE169309) [40, 41]. Availability of all other data is described in Table 1.
